# Exploring sleep heart rate variability: linear, nonlinear, and circadian rhythm perspectives

**DOI:** 10.3389/fvets.2024.1386425

**Published:** 2024-04-11

**Authors:** Mizuki Hasegawa, Mayuko Sasaki, Yui Umemoto, Rio Hayashi, Akari Hatanaka, Marino Hosoki, Ahmed Farag, Katsuhiro Matsuura, Tomohiko Yoshida, Kazumi Shimada, Lina Hamabe, Ken Takahashi, Ryou Tanaka

**Affiliations:** ^1^Department of Veterinary Surgery, Tokyo University of Agriculture and Technology, Tokyo, Japan; ^2^Yokohama Isogo Animal Hospital, Yokohama, Kanagawa, Japan; ^3^Department of Small Animal Clinical Sciences, College of Veterinary Medicine University of Florida, Gainesville, FL, United States; ^4^Department of Small Animal Medical Center, Obihiro University of Agriculture and Veterinary Medicine, Obihiro, Hokkaido, Japan; ^5^Department of Pediatrics, Juntendo University, Urayasu Hospital, Chiba, Japan

**Keywords:** autonomic balance, parasympathetic nerves, sympathetic nerves, early detection, daytime activities

## Abstract

**Background:**

Heart rate variability (HRV) is believed to possess the potential for disease detection. However, early identification of heart disease remains challenging, as HRV analysis in dogs primarily reflects the advanced stages of the disease.

**Hypothesis/objective:**

The aim of this study is to compare 24-h HRV with sleep HRV to assess the potential utility of sleep HRV analysis.

**Animals:**

Thirty healthy dogs with no echocardiographic abnormalities were included in the study, comprising 23 females and 7 males ranging in age from 2 months to 8 years (mean [standard deviation], 1.4 [1.6]).

**Methods:**

This study employed a cross-sectional study. 24-h HRV and sleep HRV were measured from 48-h Holter recordings. Both linear analysis, a traditional method of heart rate variability analysis, and nonlinear analysis, a novel approach, were conducted. Additionally, circadian rhythm parameters were assessed.

**Results:**

In frequency analysis of linear analysis, the parasympathetic index nHF was significantly higher during sleep compared to the mean 24-h period (mean sleep HRV [standard deviation] vs. mean 24 h [standard deviation], 95% confidence interval, *p* value, r-family: 0.24 [0.057] vs. 0.23 [0.045], 0.006–0.031, *p* = 0.005, *r* = 0.49). Regarding time domain analysis, the parasympathetic indices SDNN and RMSSD were also significantly higher during sleep (SDNN: 179.7 [66.9] vs. 156.6 [53.2], 14.5–31.7, *p* < 0.001, *r* = 0.71 RMSSD: 187.0 [74.0] vs. 165.4 [62.2], 13.2–30.0, *p* < 0.001, *r* = 0.70). In a geometric method of nonlinear analysis, the parasympathetic indices SD1 and SD2 showed significantly higher values during sleep (SD1: 132.4 [52.4] vs. 117.1 [44.0], 9.3–21.1, *p* < 0.001, *r* = 0.70 SD2: 215.0 [80.5] vs. 185.9 [62.0], 17.6–40.6, *p* < 0.001, *r* = 0.69). Furthermore, the circadian rhythm items of the parasympathetic indices SDNN, RMSSD, SD1, and SD2 exhibited positive peaks during sleep.

**Conclusion:**

The findings suggest that focusing on HRV during sleep can provide a more accurate representation of parasympathetic activity, as it captures the peak circadian rhythm items.

## 1 Introduction

Heart rate variability (HRV) refers to the variation in time between successive heartbeats, or RR intervals, caused by the changes in autonomic nerve stimulation to the sinus node (1). HRV analysis in human medicine has emerged as a valuable analytical tool for early disease detection and prognostic prediction across various conditions. For example, in COVID-19, a decrease in HRV has been observed to predict cardiac injury earlier than myocardial markers, suggesting that its early detection could potentially enhance patient prognosis (2). In the context of cardiovascular abnormalities, studies have suggested that decreased resting HRV in children conceived through assisted reproduction techniques may predispose them to premature cardiovascular aging (3). Abnormal HRV parameters have also been suggested to be associated with the development of congestive heart failure in asymptomatic individuals (4). On the other hand, recent findings in dogs suggest that combination therapy involving pimobendan, furosemide, and enalapril restores normal autonomic nervous system activity in dogs with myxomatous mitral valve degeneration (MMVD) stage C (5). It has been reported that both sympathetic and parasympathetic tone are altered in dogs with mitral valve disease before clinical signs appear, as demonstrated by the using of short-term HRV analysis (6). Some reports have also evaluated the influence of the dog-owner relationship on emotional reactivity in dogs and whether medication can positively affect stress indicators (7, 8). In dogs, it has been utilized to assess cardiac autonomic balance in therapy, disease assessment, and behavioral research (5–7). Other have been reported in a variety of areas, such as assessing stress levels related to animal welfare, evaluating intraoperative analgesia and nociceptive balance, and assessing intraoperative pain to improve postoperative care (9–11). In all of this cases there is a predominant sympathetic tone and a consequent endocrine response that directly influences heart rate and, therefore, HRV (12–15).

Several reports aiming early detection of cardiac disease in dogs have shown that sympathetic indices of HRV parameters increase as heart disease progresses (16). Respiratory arrhythmias, characterized by variations in the heart rate that are synchronized with the respiratory cycle, commonly occur during parasympathetic (vagal) tone. Particularly in the presence of heart disease, these respiratory arrhythmias can be a factor in assessing the progression of the condition, as they may diminish or become less prominent as the disease progresses (17). On the other hand, canine respiratory arrhythmias can complicate HRV analysis. Normal RR interval variability in dogs during sleep and rest can reach as high as 77%, while extrasystoles have been observed to exhibit variability ranging from 50 to 60%. This variability can make them challenging to distinguish in conventional linear analysis, which may result in the exclusion of data that could potentially contain valuable information for disease identification (16, 18, 19). Therefore, some reports indicate that HRV analysis in dogs may only reflect only advanced disease, posing challenges for its use in early detection and prognosis prediction unlike in human medicine (11). Additionally, in humans, daytime activity is also a factor that disrupts heart rate variability (20). Even in the same individual, unrestricted activity can vary from day to day, unpredictably affecting 24-h HRV (21). In recent years, sleep has been proposed as a time-efficient measure of HRV that is less susceptible to environmental factors than daytime measurements (22). Although the relationship between sleep HRV and cardiovascular events in humans is emerging, there is limited data specifically on sleep HRV in dogs.

Based on the above, we hypothesized that there is a difference between sleep HRV and 24-h HRV in dogs. We focused on the sleep period, during which the parasympathetic nervous system is dominant, and respiratory arrhythmia is high, unaffected by daytime activities. The aim of this study is to compare 24-h HRV with sleep HRV to assess the potential utility of sleep HRV analysis. A comparison between 24-h HRV and sleep HRV was conducted using both conventional analysis method, such as linear analysis, and a novel analysis method, nonlinear analysis, which has been shown to be an indicator with high specificity, sensitivity, and diagnostic accuracy for identifying dogs at risk of death (23).

## 2 Materials and methods

This is a cross-sectional study.

### 2.1 Animals

Dogs for the study were selected from those brought to the Department of Dog & Cat Pediatric Hospital in Tokyo, Japan. Healthy dogs were chosen from among those admitted to the hospital for pet boarding or temporary dog care. Additionally, experimental Beagle dogs from our laboratory at Kitayama Labes in Nagano, Japan, were enrolled between August 2018 and January 2023. G*Power (The G*Power Team, G*Power 3.1.9.7 version, Germany) was used to calculate sample sizes. To adapt a paired *t*-test, we set *α* = 0.05, 1-β = 0.8, and effect size (d-family) = 0.5. The sample size was calculated to require at least 34 cases, so efforts were made to collect dogs for the study as a target value. Healthy dogs with normal physical examination and echocardiography were selected as the test subjects for this study. Dogs with obvious pain on physical examination were excluded. Puppies weighing less than 1.0 kg and too small to be fitted with a Holter electrocardiograph were also excluded from the study. All dog owners provided their consent for their pets to participate in the study. Experimental dogs were handled according to the guidelines established by the Institutional Animal Care and Use Committee of the TUAT (Approval number: R05-140).

### 2.2 Holter monitoring

**Figure 1:**
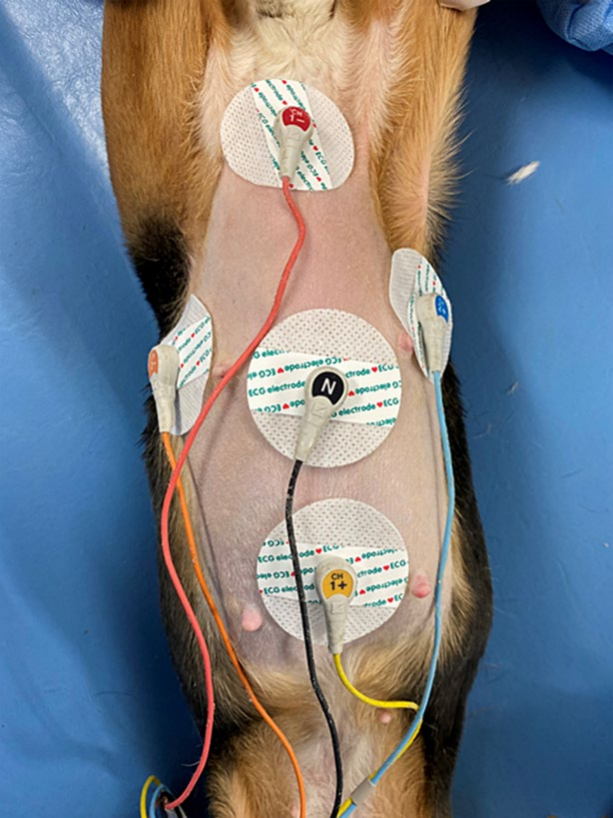
The M-X induction method and the R-L induction method. CH1- (red) electrodes placed on the manubrium of the sternum, CH1+ (yellow) on the xiphoid process, CH2- (orange) on the right 5th ~ 6th intercostal space, CH2+ (blue) on the left 5th ~ 6th intercostal space, and a ground electrode (black) in the middle.

**Figure 2:**
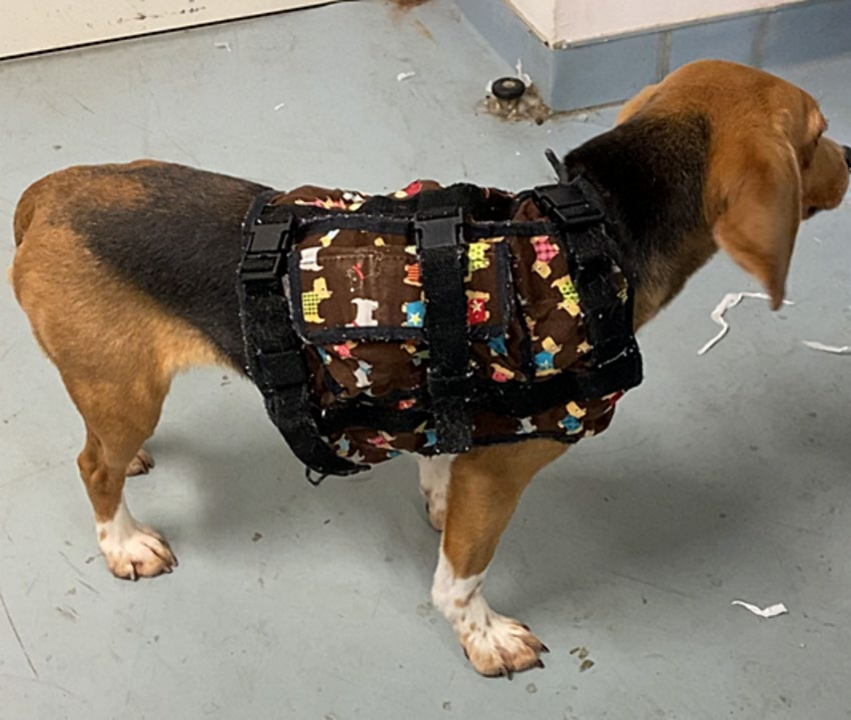
A vest to secure the Holter recorder and leads to the dog.

The Holter electrocardiograph used in this study was manufactured by NIHON KOHDEN CORPORATION (RAC-5203, Japan). Prior to electrode placement, the dogs’ thoraxes were shaved vertically from the sternal scape to the xiphoid process and horizontally around the fifth and sixth intercostal spaces and cleaned with alcohol. Disposable ECG electrodes (XUNDA BRAND, China) were positioned using the M-X induction method and the R-L induction method, which is perpendicular to the M-X method. Subsequently, the induction cords were attached to the electrodes, CH1- (red) electrodes placed on the manubrium of the sternum, CH1+ (yellow) on the xiphoid process, CH2- (orange) on the right 5th ~ 6th intercostal space, CH2+ (blue) on the left 5th ~ 6th intercostal space, and a ground electrode (black) in the middle (24) (Figure 1). Both M-X and R-L leads were recorded. To secure the Holter recorder and leads to the dog, an elastic bandage and a vest utilized (Figure 2). Holter electrocardiogram (ECG) measurements were conducted by veterinarians and clinical laboratory technicians in the TUAT laboratory for 48 h period, during which the animals were allowed free movement within the enclosure.

### 2.3 Heart rate variability

For the 48-h Holter ECG measurements, the period from 12 p.m. on the first night to 12 a.m. on the second night was designated for 24-h HRV, while the period from 12 p.m. on the first night to 8 a.m. on the second day was earmarked for analysis as sleep HRV. HRV analysis was conducted using the Juntendo University algorithm with MATLAB (MathWorks, R2022a, United States). Both traditional linear analysis and a newer nonlinear analysis method were employed under the following conditions.

#### 2.3.1 Linear heart rate variability

HRV variables for frequency analysis include total power (TP, 0–0.4 Hz), ultra low frequency (ULF, 0–0.00333 Hz), very low frequency (VLF, 0.00333–0.04 Hz), low frequency (LF, 0.04–0.15 Hz), and high frequency (HF, 0.15–0.4 Hz). The parameters utilized in this study were normalized high frequency (nHF) and LF/HF ratio. Normalization eliminates much of the significant within-subject and between-subject variation, resulting in increased reproducibility. Therefore, HF and LF were normalized using the equations nHF = HF / (LF + HF) and normalized low frequency (nLF) = LF / (LF + HF) (25). Regarding time domain analysis in linear analysis, standard deviation on NN intervals (SDNN) and root mean squared of successive RR intervals (RMSSD) were employed. SDANN, SDNN index and pNN50 were not included in the analysis for the following reasons (26). (1) SDANN is correlated with SDNN and is generally considered redundant. (2) SDNN index only estimates variability due to factors affecting HRV within a 5 min period. (3) RMSSD typically provides a better assessment of respiratory sinus arrhythmia (RSA) and most researchers prefer it over pNN50.

#### 2.3.2 Nonlinear heart rate variability

For the nonlinear analysis, both geometric and fractal analyses were employed. Geometric analysis involved plotting a Poincaré plot by graphing every RR interval against the prior interval, thus creating a scatter plot. This plot can be analyzed by fitting an ellipse to the plotted points. The standard deviation of the distance of each point from the y = x axis was measured as SD1 (width of ellipse), While the standard deviation of the distance of each point from y = x + mean R-R interval was measured as SD2 (length of ellipse) (26–31). The ratio SD1/SD2 was measured to assess autonomic balance. Since the healthy heartbeat interval are complex and variable, detrended fluctuation analysis (DFA) was utilized for fractal analysis. DFA quantifies the correlative properties in non-stationary physiological series by examining correlations between consecutive RR intervals (32, 33).

#### 2.3.3 Circadian rhythm

Circadian rhythms were measured to identify the maximum peak for each item. The entire 24-h normal beat RR interval data were divided into 5-min segments for circadian rhythm analysis. HRV circadian rhythm items were fitted to a cosine periodic function and measured (34). These measurements included nHF, LF/HF, SDNN, RMSSD, SD1, SD2, SD1/SD2, and DFA.

### 2.4 Statistical analysis

Statistical analyses were conducted using R software (R Development Core Team, version 4.1.0, New zealand). The significance level set at *p* < 0.05. A difference test was adapted to examine the difference between 24-h HRV and sleep HRV. Normality was confirmed using the Shapiro–Wilk test. If the distribution followed a normal distribution, a paired t-test was used. Conversely, if the distribution did not follow a normal distribution, the Wilcoxon signed-rank sum test was applied. Parametric data are presented as means and standard deviations, with 95% confidence intervals also calculated. Nonparametric data are presented as median and interquartile range. The effect size was calculated using R-family. HRV circadian rhythm times were quantified.

## 3 Results

### 3.1 Animals

A total of 30 dogs were included in the present study, comprising 23 females and 7 males, with ages ranging from 2 months to 8 years (mean [standard deviation], 1.4 [1.6]), and weights ranging from 1.7 to 9.3 kg (5.6 [2.9]). The breeds included Beagles (*n* = 16), Chihuahuas (*n* = 4), Miniature Schnauzers (*N* = 2), Mongrels (*n* = 3), Miniature Pinscher (*n* = 1), Yorkshire Terrier (*n* = 1), Maltese (*n* = 1), Border Collie (*n* = 1), and Miniature Dachshund (*n* = 1). No abnormal rhythm findings that would affect HRV were detected on the Holter ECG.

### 3.2 Linear heart rate variability

Linear HRV data were summarized in Table 1. Frequency analysis nHF was significantly higher in sleep HRV compared to in 24-h HRV (mean [standard deviation], 95% confidence interval, *p* value, r-family: 0.24 [0.057] vs. 0.23 [0.045], 0.006–0.031, *p* = 0.005, *r* = 0.49). Meanwhile, there was no statistically significant difference in LF/HF between 24-h HRV and sleep HRV (0.87 [0.38] vs. 0.84 [0.49], −0.11-0.15, *p* = 0.72, *r* = 0.067). In time domain analysis, both SDNN and RMSSD were significantly higher in sleep HRV than in 24-h HRV (SDNN: 179.7 [66.9] vs. 156.6 [53.2], 14.5–31.7, *p* < 0.001, *r* = 0.71) (RMSSD: 187.0 [74.0] vs. 165.4 [62.2], 13.2–30.0, *p* < 0.001, *r* = 0.70).

**Table 1:** Heart rate variability variables during 24 h and sleep in 30 dogs.

Indices	Units	24 h HRV		Sleep HRV		95% Confidence interval	*p* value	r-family
		Mean (SD)	Median (range)	Mean (SD)	Median (range)			
nHF	ms^2^	0.23 (0.045)	–	0.24 (0.057)	–	0.006–0.031	0.005*	0.49
LF/HF	ms^2^	0.87 (0.38)	–	0.84 (0.49)	–	−0.11 - 0.15	0.72	0.067
SDNN	ms	156.6 (53.2)	–	179.7 (66.9)	–	14.5–31.7	< 0.001*	0.71
RMSSD	ms	165.4 (62.2)	–	187.0 (74.0)	–	13.2–30.0	< 0.001*	0.7
SD1	ms	117.1 (44.0)	–	132.4 (52.4)	–	9.3–21.2	< 0.001*	0.7
SD2	ms	185.9 (62.0)	–	215.0 (80.5)	–	17.6–40.6	< 0.001*	0.69
SD1/SD2	%	–	0.63 (0.59–0.66)	–	0.60 (0.54–0.65)	–	0.43	0.041
DFA		0.71 (0.11)	–	0.71 (0.12)	–	−0.024–0.040	0.62	0.092

Data are expressed as mean, standard deviation, and median, range. Asterisks (*) are used to compare significance between 24 h HRV and sleep HRV (*p < 0.05). nHF, SDNN, RMSSD, SD1, and SD2 were significantly higher for sleep HRV. For abbreviations of HRV variables, nHF, normalized high frequency; LF, low frequency; SDNN, Standard deviation of NN intervals; RMSSD, Root mean square of successive RR interval differences; SD1, Poincaré plot standard deviation perpendicular the line of identity; SD2, Poincaré plot standard deviation along the line of identity; DFA, Detrended fluctuation analysis.

### 3.3 Nonlinear heart rate variability

Nonlinear HRV data were summarized in Table 1. In the geometric analysis, both SD1 and SD2 were significantly higher sleep HRV than 24-h HRV (SD1: 132.4 [52.4] vs. 117.1 [44.0], 9.3–21.1, *p* < 0.001, *r* = 0.70) (SD2: 215.0 [80.5] vs. 185.9 [62.0], 17.6–40.6, *p* < 0.001, *r* = 0.69). However, there was no significant difference in SD1/SD2 between 24-h HRV and sleep HRV (median [interquartile range], 0.63 [0.59–0.66] vs. 0.60 [0.54–0.65], *p* = 0.43, *r* = 0.041). Additionally, DFA in fractal analysis showed no statistically significant difference between 24-h HRV and sleep HRV (0.71 [0.11] vs. 0.71[0.12], −0.024-0.040, *p* = 0.62, *r* = 0.092).

### 3.4 Circadian rhythm

**Figure 3:**
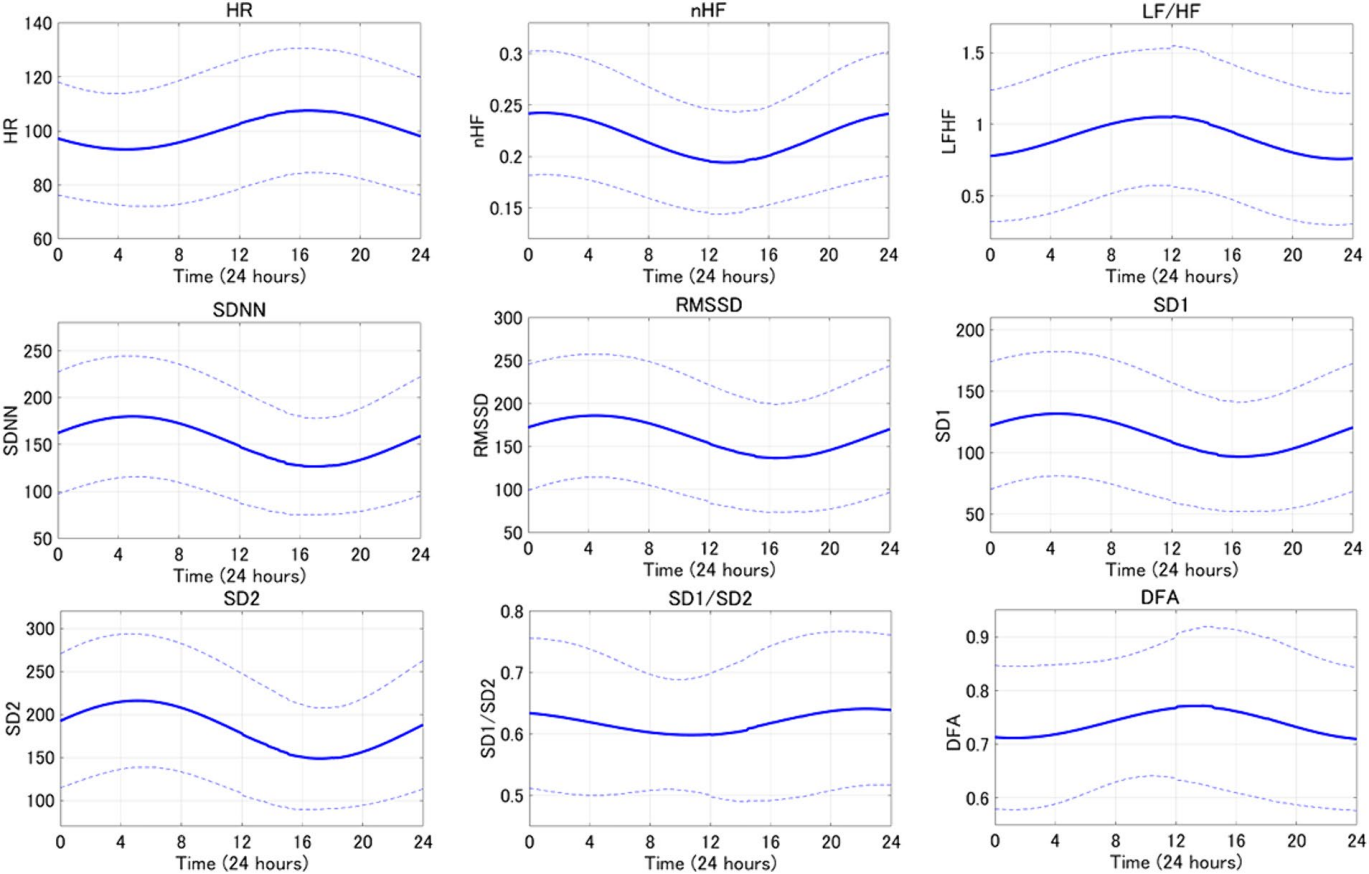
The averaged circadian rhythm averaging curve for the 24-h HRV index is shown. Bold lines indicate mean curves and dotted lines indicate standard deviations.

**Figure 4:**
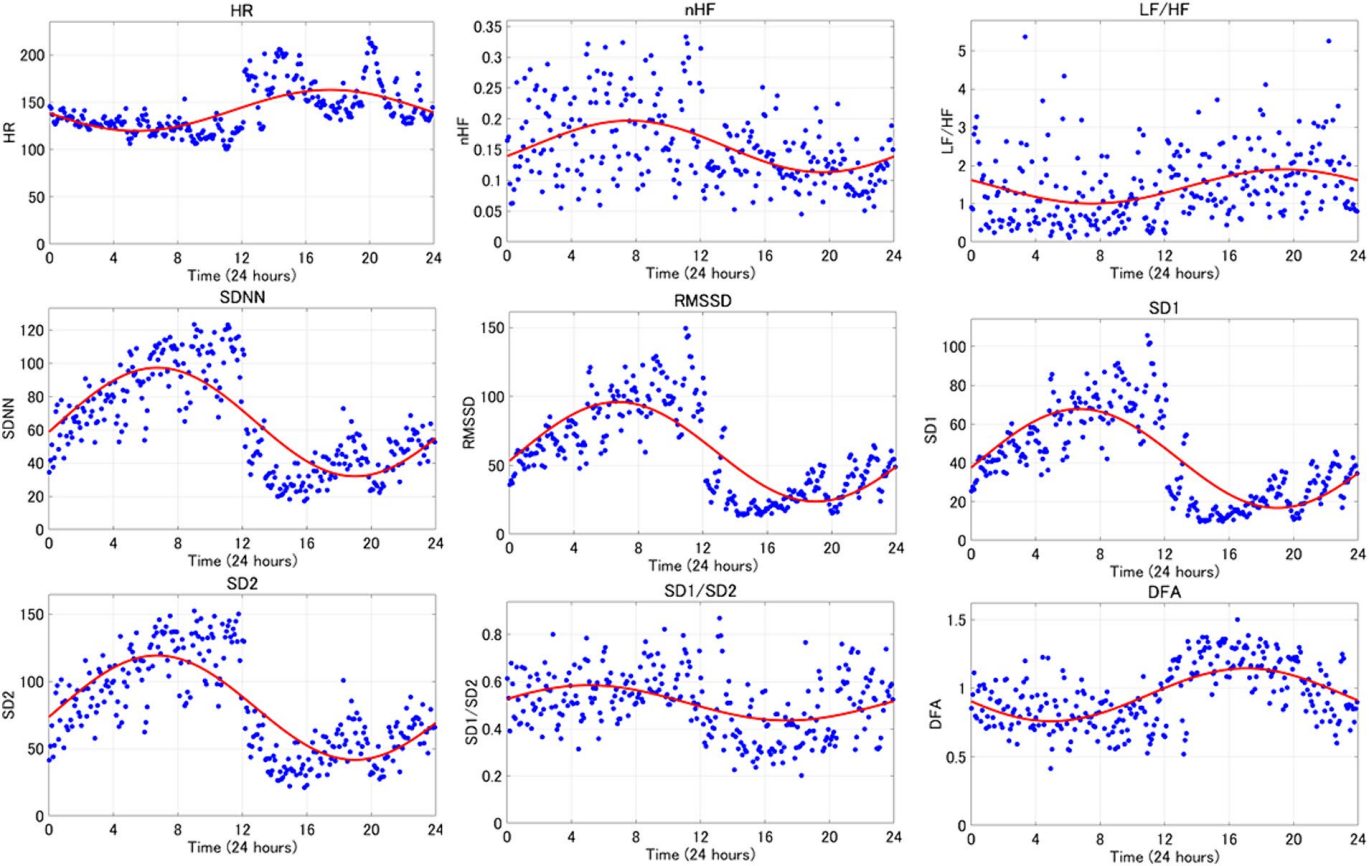
The circadian rhythm items of the 24-h HRV index are shown. This is one representative example. The red curve represents the circadian rhythm and the blue dots represent the 5-min readings for each indicator.

An average example of circadian rhythm elements for HRV indicators is shown in Figure 3, while a representative example is illustrated in Figure 4. The parasympathetic indicators SDNN (mean [standard deviation]: 6.42 [5.07]), RMSSD (7.45 [6.41]), SD1 (7.45 [6.41]), and SD2 (5.8 [3.82]) exhibited positive peaks during sleep HRV. However, the positive peak for nHF (9.92 [8.06]), a parasympathetic index, was observed to fall outside the range defined as sleep HRV.

## 4 Discussion

### 4.1 Brief summary

One of the main objectives of the present study was to compare 24-h HRV with sleep HRV to delineate the differences. The findings of the present study suggest that the parasympathetic indices nHF, SDNN, RMSSD, SD1, and SD2 predominantly reflect parasympathetic activity during sleep. Moreover, as the positive peaks of the circadian rhythm elements of the parasympathetic indices fall within the range defined as HRV during sleep, HRV during sleep can serve as an indicator of the peak parasympathetic activity during the day. These results support the hypothesis that differences exist between 24 h HRV and sleep HRV.

### 4.2 Comparison with previous studies

Previous studies in humans have suggested that parasympathetic activity increases during the night with the circadian rhythm components of the HRV parasympathetic index exhibiting a positive peak during nighttime (34). The data presented in the present study suggest that sleep HRV may be useful metric in dogs, as a valuable analytical tool for early detection and prognosis of various diseases, as they exhibit similar changes to those observed in humans. This study distinguishes itself from previous studies by utilizing circadian rhythms to identify the maximum peak of circadian rhythm elements of parasympathetic indices within a 24-h period focusing specifically on the measurement range of sleep HRV. Rasmussen et al. (35) defined sleep HRV as the period starting from 30 min after the dog enters sleep and extending to 6 h. Blake et al. (28) also defined resting HRV as the period from 0:00 to 6:00 and activity HRV as the period from 12:00 to 18:00. Since both measurements are based on activity records, the measurement times are back and forth. Therefore, it would enhance the external validity of the results if the peak time of the HRV circadian rhythm item could be utilized as a reference when evaluating the HRV index.

### 4.3 Possible explanation and implications

The nHF observed in the frequency analysis was consistent with expectations, showing high values during HRV in sleep. However, the circadian rhythm item of the parasympathetic index deviated from expectations by falling outside the range defined in this study as HRV during sleep. Identifying the peak time of parasympathetic activity proves challenging even when utilizing circadian rhythms due to significant individual variation in nHF and the discrepancy between the average peak derived from each case and the peak calculated from the average curve. nHF demonstrates respiratory variability due to interference from the respiratory center and reflexive input from the periphery, which is transmitted to the sinus node to become HF. Therefore, it is plausible that nHF may not be considered a pure indicator of the cardiac vagus nerve activity (20, 36). SDNN in time domain analysis is known to be influenced by the duration of analysis. In short-term recordings, the primary source of SDNN variability is parasympathetically mediated RSA, whereas in 24-h recordings, sympathetic nerves contribute significantly to SDNN (20, 31). Therefore, it is expected that the difference in analysis time between 24-h HRV and 1-h sleep HRV would impact SDNN values. RMSSD is considered a superior indicator of parasympathetic activity compared to SDNN. However, RMSSD is less affected by respiration than SDNN, but more influenced by RSA (37). RSA also depend on a number of control mechanisms due to interference from cardiovascular centers in the medulla oblongata, the degree of lung distension, and reflexive input from right atrial wall distension (38). Therefore, interpretations other than cardiac vagal activity must be considered, but this potential influence is rarely taken into account in linear analysis methods (39). Consequently, it was hypothesized that dogs with physiological respiratory arrhythmias might be affected by this phenomenon. Linear analysis of HRV during sleep in dogs may be susceptible to several biases, including those related to respiration, duration, and respiratory arrhythmia. Therefore, utilizing linear analysis for HRV measurement in dogs may not be an appropriate analytical method.

On the contrary, nonlinear analysis is an analytical method that circumvents the drawbacks associated with linear analysis. While linear analysis entails examining the time series of RR intervals obtained. Life is inherently nonlinear, meaning that the relationship between variables cannot be plotted as a straight line. Nonlinear analysis serves as a method of assessing the unpredictability of a time series and reveals correlations when the complexity arises from the same underlying process (26). In humans, nonlinear analysis has attracted attention due to its potential to predict the onset of heart failure (4). Additionally, HRV measurement during sleep, which is less susceptible to environmental factors compared to daytime measurements, has been proposed (22). Therefore, considering that SD1 and SD2 in this study were nonlinear analyses and showed significant increases during sleep compared to the 24-h period, and that the maximum peak of circadian rhythm items occurred during sleep, we believe that future investigations into heart disease in dogs utilizing sleep HRV and nonlinear analyses could be advantageous for early detection of heart disease mirroring approaches used in humans.

### 4.4 Limitations

One limitation of this study is the lack of consideration for breed differences, as well as the wide age range of the subjects, spanning from 2 months to 8 years. In humans, studies such as those by Bonnemeier et al. (40) have shown that HRV indices experience the most significant decline between the ages of 20 and 30. Additionally, Almeida-Santos et al. also reported that RMSSD decreases between the ages of 40 and 60, followed by an increase after the age of 70 (41). Therefore, age may have influenced the findings of the present study. As a perspective for future studies to clarify the effects of breed and age on HRV in dogs, it may be necessary to equalize breeds or differentiate between small, medium, and large dogs, or to conduct HRV studies by age stratification. Another limitation is the inclusion of brachycephalic breeds. It has been suggested that brachycephalic breeds may exhibit higher cardiac vagal activity compared to non-brachycephalic breeds (42). Furthermore, the heterogeneity of the rearing environment of the experimental animals is another limitation. If future studies are conducted with domestic dogs living in human households, the circadian rhythm may be influenced by the human life rhythm, potentially impacting HRV measurements. In practice, we are investigating the early detection of doxorubicin-induced myocardial damage. If myocardial damage is detected before irreversibility, it can be treated. Sleep HRV may detect smaller myocardial changes.

## 5 Conclusion

In conclusion, SDNN, RMSSD, SD1, and SD2 significantly reflected parasympathetic activity during sleep. Focusing on HRV during sleep enables us to capture the maximum peak of the circadian rhythm items of the parasympathetic index of HRV and more accurately represents parasympathetic activity than 24-h HRV.

## Data availability statement

The original contributions presented in the study are included in the article/supplementary material, further inquiries can be directed to the corresponding author.

## Ethics statement

The animal studies were approved by the Institutional Animal Care and Use Committee of the TUAT. The studies were conducted in accordance with the local legislation and institutional requirements. Written informed consent was obtained from the owners for the participation of their animals in this study.

## Author contributions

MHa: Conceptualization, Data curation, Formal analysis, Investigation, Methodology, Project administration, Validation, Visualization, Writing – original draft, Writing – review & editing. MS: Data curation, Project administration, Writing – review & editing. YU: Writing – review & editing, Data curation. RH: Writing – review & editing, Data curation. AH: Writing – review & editing, Data curation. MHo: Writing – review & editing, Data curation. AF: Writing – review & editing, Data curation. KM: Writing – review & editing. TY: Writing – review & editing. KS: Writing – review & editing. LH: Writing – review & editing. KT: Conceptualization, Investigation, Methodology, Software, Visualization, Writing – review & editing. RT: Conceptualization, Investigation, Methodology, Project administration, Supervision, Validation, Writing – review & editing.

## References

[1] Santilli RA Moise NS Pariaut R Perego M. Electrocardiography of the dog and cat. Milano: Edra S.p.A (2018). 77 p.

[2] Chengfen Y Jianguo L Zhiyong W Yongle Z Lei X. Decreased heart rate variability in COVID-19. Intensive Care Res. (2023) 3:87–91. doi: 10.1007/s44231-022-00024-1, PMID: 36471860 PMC9713139

[3] Ikram M Morten AVL Ann VL Louise LA Niels-Henrik H-R Gorm G. Cardiovascular autonomic nervous function in children conceived by assisted reproductive technology with frozen or fresh embryo transfer. Am J Physiol Heart Circ Physiol. (2024) 326:H216–22. doi: 10.1152/ajpheart.00680.2023 37999646

[4] Vaiibhav NP Brian RP Rohan KB David LB Diane GI Phyllis KS. Association of holter-derived heart rate variability parameters with the development of congestive heart failure in the cardiovascular health study. JACC. Heart Failure. (2017) 5:424–31. doi: 10.1016/j.jchf.2016.12.015 PMC5851278 28396041

[5] Prapawadee P Nakkawee S Pakit B Robert LH Anusak K. Impact of a combination of pimobendan, furosemide, and enalapril on heart rate variability in naturally occurring, symptomatic, myxomatous mitral valve degeneration dogs. BMC Vet Res. (2023) 19:201. doi: 10.1186/s12917-023-03770-6, PMID: 37821927 PMC10568857

[6] Mathieu B Vincent P Geoffroy S Pedro MV José HR Peter V. Association between nocturnal heart rate variability and incident cardiovascular disease events: the HypnoLaus population-based study. Heart Rhythm. (2022) 19:632–9. doi: 10.1016/j.hrthm.2021.11.033, PMID: 34864166

[7] Sanni S Heini T Aija K Antti V Veikko S Outi V. Dog–owner relationship, owner interpretations and dog personality are connected with the emotional reactivity of dogs. Animals. (2022) 12:1338. doi: 10.3390/ani12111338 35681804 PMC9179432

[8] Alysia BGH Hannah EF Darren WL Tammie K. A single dose of cannabidiol (CBD) positively influences measure of stress in dogs during separation and car travel. Front Vet Sci. (2023) 10:1112604. doi: 10.3389/fvets.2023.1112604, PMID: 36908527 PMC9992179

[9] Turini L Bonelli F Lanata A Vitale V Nocera I Sgorbini M. Validation of a new smart textiles biotechnology for heart rate variability monitoring in sheep. Front Vet Sci. (2022) 22:1018213. doi: 10.3389/fvets.2022.1018213 PMC9722759 36483489

[10] Mansour C Merlin T Bonnet-Garin JM Chaaya R Mocci R Ruiz CC. Evaluation of the parasympathetic tone activity (PTA) index to assess the analgesia/nociception balance in anaesthetized dogs. Res Vet Sci. (2017) 115:271–7. doi: 10.1016/j.rvsc.2017.05.009, PMID: 28575801

[11] Hernández-Avalos I Valverde A Ibancovichi-Camari JA Sanchez-Aparicio P Recillas-Morales S Rodríguez-Velázquez D. Clinical use of the parasympathetic tone activity index as a measurement of postoperative analgesia in dogs undergoing ovariohysterectomy. J Vet Res. (2021) 65:117–23. doi: 10.2478/jvetres-2021-0004, PMID: 33817404 PMC8009586

[12] Eberhard VB Jan L Gérard D Sven H Le C Jeremy M. Heart rate variability as a measure of autonomic regulation of cardiac activity for assessing stress and welfare in farm animals — a review. Physiol Behav. (2007) 92:293–316. doi: 10.1016/j.physbeh.2007.01.007, PMID: 17320122

[13] Hernández-Avalos I Mota-Rojas D Mendoza-Flores JE Casas-Alvarado A Flores-Padilla K Miranda-Cortes AE. Nociceptive pain and anxiety in equines: physiological and behavioral alterations. Vet World. (2021) 14:2984–95. doi: 10.14202/vetworld.2021.2984-2995, PMID: 35017848 PMC8743789

[14] Nancy G Zuzanna Z Annabel W Ross H Valeria M. Heart rate variability (HRV) as a way to understand associations between the autonomic nervous system (ANS) and affective states: a critical review of the literature. Int J Psychophysiol. (2023) 192:35–42. doi: 10.1016/j.ijpsycho.2023.08.001 37543289

[15] Gaidica M Dantzer B. Quantifying the autonomic response to stressors—one way to expand the definition of “stress” in animals. ICB. (2020) 60:113–25. doi: 10.1093/icb/icaa009, PMID: 32186720

[16] Oliveira MS Muzzi RAL Araújo RB Muzzi LAL Ferreira DF Nogueira R. Heart rate variability parameters of myxomatous mitral valve disease in dogs with and without heart failure obtained using 24-hour Holter electrocardiography. Vet Rec. (2012) 170:622. doi: 10.1136/vr.100202, PMID: 22645158

[17] Clay AC. Heart rate variability. J Small Anim Pract. (1998) 28:1409–27. doi: 10.1016/S0195-5616(98)50129-5 10098245

[18] Clay AC Michelle W Clay A. Effect of severity of myocardial failure on heart rate variability in Doberman pinschers with and without echocardiographic evidence of dilated cardiomyopathy. JAVMA. (2001) 219:1084–8. doi: 10.2460/javma.2001.219.1084, PMID: 11700705

[19] Alan WS Kathryn MM. Assessment of heart rate variability in boxers with arrhythmogenic right ventricular cardiomyopathy. JAVMA. (2004) 224:534–7. doi: 10.2460/javma.2004.224.534 14989545

[20] David AG Nick D. Heart rate variability during exercise: a comparison of artefact correction methods. J Strength Cond Res. (2018) 32:726–35. doi: 10.1519/JSC.0000000000001800 29466273

[21] Bernardi L Valle F Coco M Calciati A Sleight P. Physical activity influences heart rate variability and very-low-frequency components in Holter electrocardio grams. Cardiovasc Res. (1996) 32:234–7. doi: 10.1016/0008-6363(96)00081-8, PMID: 8796109

[22] David H Prisca E Ximena O Robert R Matthias W Peter A. Reproducibility of heart rate variability is parameter and sleep stage dependent. Front Physiol. (2018) 10:1100. doi: 10.3389/fphys.2017.01100 PMC5767731 29367845

[23] Martinello L Romão FG Godoy MF Machado LHA Tsunemi MH Lourenço MLG. Study of autonomic modulation by non-linear analysis of heart rate variability in different age groups and analysis of health status, disease and risk of death in dogs. Pol J Vet Sci. (2023) 26:581–90. doi: 10.24425/pjvs.2023.148278, PMID: 38088302

[24] Uchino T. About long-term ECG recording methods in dogs (in Japanese). Jpn J Electrocardiology. (2001) 21:45–51. doi: 10.5105/jse.21.Suppl1_45

[25] Robert LB. Interpretation of normalized spectral heart rate variability indices in sleep research: a critical review. Sleep. (2007) 30:913–9. doi: 10.1093/sleep/30.7.913, PMID: 17682663 PMC1978375

[26] Shaffer F Ginsberg JP. An overview of heart rate variability metrics and norms. Front Public Health. (2017) 28:258. doi: 10.3389/fpubh.2017.00258 PMC5624990 29034226

[27] Moïse NS Gladuli A Hemsley SA Otani NF. “Zone of avoidance”: RR interval distribution in tachograms, histograms, and Poincaré plots of a boxer dog. J Vet Cardiol. (2010) 12:191–6. doi: 10.1016/j.jvc.2010.07.001, PMID: 21036115 PMC3184837

[28] Blake RR Shaw DJ Culshaw GJ Martinez-Pereira Y. Poincare’ plots as a measure of heart rate variability in healthy dogs. J Vet Cardiol. (2018) 20:20–32. doi: 10.1016/j.jvc.2017.10.006, PMID: 29277470

[29] Moïse NS Brewer FC Flanders WH Kornreicha BG Otani NF. Insights into sinus arrhythmia of the dog: acetylcholine perfusion of canine right atrium results in beat-to-beat patterns that mimic sinus arrhythmia supporting exit block in the sinoatrial conduction pathways. Vet J. (2021) 272:105651. doi: 10.1016/j.tvjl.2021.105651, PMID: 33745806

[30] Giovanni R Carlo G Helen P Marco BT. Lorenz plot analysis in dogs with sinus rhythm and Tachyarrhythmias. Animals. (2021) 11:1645. doi: 10.3390/ani11061645 34206036 PMC8228210

[31] Flanders WH Moïse NS Pariaut R Sargent J. The next heartbeat: creating dynamic and histographic Poincaré plots for the assessment of cardiac rhythms. J Vet Cardiol. (2022) 42:1–13. doi: 10.1016/j.jvc.2022.04.003, PMID: 35662023

[32] Peng CK Havlin S Stanley HE Goldberger AL. Quantification of scaling exponents and crossover phenomena in nonstationary heartbeat time series. Chaos. (1995) 5:82–7. doi: 10.1063/1.166141, PMID: 11538314

[33] Peng CK Havlin S Hausdorff JM Mietus J Stanley HE Goldberger AL. Fractal mechanisms and heart rate dynamics: long-range correlations and their breakdown with disease. J Electrocardiol. (1996) 28:59–65. doi: 10.1016/s0022-0736(95)80017-4 8656130

[34] Xian L Michele LS Sol RC Fan H Deborah LW Peter AJ. The circadian pattern of cardiac autonomic modulation in a middle-aged population. Clin Auton Res. (2011) 21:143–50. doi: 10.1007/s10286-010-0112-4, PMID: 21240538 PMC3093547

[35] Rasmussen CE Falk T Zois NE Moesgaard SG Haggstrom J Pedersen HD. Heart rate, heart rate variability, and arrhythmias in dogs with myxomatous mitral valve disease. J Vet Intern Med. (2012) 26:76–84. doi: 10.1111/j.1939-1676.2011.00842.x, PMID: 22151356

[36] Paul G Edwin WT. Toward understanding respiratory sinus arrhythmia: relations to cardiac vagal tone, evolution and biobehavioral functions. Biol Psychol. (2007) 74:263–85. doi: 10.1016/j.biopsycho.2005.11.014, PMID: 17081672

[37] Shaffer F McCraty R Zerr CL. A healthy heart is not a metronome: an integrative review of the heart’s anatomy and heart rate variability. Front Psychol. (2014) 5:1040. doi: 10.3389/fpsyg.2014.01040, PMID: 25324790 PMC4179748

[38] Moise NS. From cell to cageside: autonomic influences on cardiac rhythms in the dogs. J Small Anim Pract. (1998) 39:460–8. doi: 10.1111/j.1748-5827.1998.tb03680.x, PMID: 9816568

[39] Ritz T. Putting back respiration into respiratory sinus arrhythmia or high-frequency heart rate variability: implication for interpretation, respiratory rhythmicity and health. Biol Phychol. (2014) 185:108728. doi: 10.1016/j.biopsycho.2023.108728 38092221

[40] Bonnemeier H Wiegand UKH Brandes A Kluge N Katus HA Richardt G. Circadian profile of cardiac autonomic nervous modulation in healthy subjects: differing effects of aging and gender on heart rate variability. J Cardiovasc Electrophysiol. (2003) 14:791–9. doi: 10.1046/j.1540-8167.2003.03078.x, PMID: 12890036

[41] Almeida-Santos MA Barreto-Filho JA Oliveira JL Reis FP da Cunha Oliveira CC Sousa ACS. Aging, heart rate variability and patterns of autonomic regulation of the heart. Arch Gerontol Geriatr. (2016) 63:1–8. doi: 10.1016/j.archger.2015.11.011, PMID: 26791165

[42] Doxey S Boswood A. Differences between breeds of dog in a measure of heart rate variability. Vet Rec. (2004) 154:713–7. doi: 10.1136/vr.154.23.713, PMID: 15214514

